# Synthesis, crystal structure and Hirshfeld surface of bis­(acetato-κ^2^*O*,*O*′)(2-benzyl-1*H*-benzimidazole-κ*N*^3^)copper(II)

**DOI:** 10.1107/S2056989025010813

**Published:** 2026-01-01

**Authors:** Gulnoza Boboyeva, Gulbeka Mamatova, Sardor Murodov, Komila Ganiyeva, Kambarali Turgunov, Bakhodir Tashkhodjaev, Shakhlo Daminova

**Affiliations:** a National University of Uzbekistan named after Mirzo Ulugbek University Street 4 Tashkent 100174 Uzbekistan; b Tashkent State Medical University, Farobiy Street, 2, Almazar district, Tashkent, 100109, Uzbekistan; c Uzbekistan-Japan Innovation Centre of Youth, University Street 2B, Tashkent, 100095, Uzbekistan; d Branch of D. I. Mendeleev University of Chemical Technology of Russia, 100142, Tashkent, Mirzo-Ulugbek District, TTZ-1, 47, Uzbekistan; e Institute of the Chemistry of Plant Substances, Uzbekistan Academy of Sciences, Mirzo Ulugbek Str. 77, Tashkent 100170, Uzbekistan; Universidad de la Repüblica, Uruguay

**Keywords:** crystal structure, dibazol, Hirshfeld surface, hydrogen bonds

## Abstract

The title copper(II) complex, [Cu(C_2_H_3_O_2_)_2_(C_14_H_12_N_2_)_2_], was synthesized and structurally characterized. It crystallizes in the monoclinic space group *P2*_1_*/n* with one mol­ecule per asymmetric unit. The Cu^2+^ ion exhibits a distorted octa­hedral (4 + 2) coordination geometry. The crystal packing is consolidated by N—H⋯O hydrogen bonds and C—H⋯π inter­actions. Hirshfeld surface analysis indicates that H⋯H, H⋯C/C⋯H and O⋯H/H⋯O contacts are the major contributors to the crystal packing.

## Abstract 2

The copper(II) complex bis­(acetato-*κ^2^O,O*′)(2-(phenyl­meth­yl)-1*H*-benzimidazol­yl)copper(II) crystallizes in the monoclinic space group *P2*_1_*/n* with the Cu^2+^ ion exhibiting a distorted octa­hedral geometry. The crystal packing features N—H⋯O and C—H⋯π inter­actions.

## 1. Chemical context

2-(Phenyl­meth­yl)-1*H*-benzimidazole, also known as dibazol (bendazol), is a benzimidazole derivative that belongs to the class of synthetic adaptogens. This organic compound is used in medicine for its immunostimulating, vasodilatory and anti­spasmodic effects (Oliynyk & Oh, 2012 ▸). Upon entering the organism, dibazol acts directly on processes in blood cells – leukocytes and platelets (Oliynyk & Oh, 2012 ▸). The structure of dibazol has been determined and the fluorescence properties of this compound were also investigated (Lü *et al.*, 2018 ▸).

At present, its coordination compounds with *d*-block metals are under investigation. We previously synthesised a number of coordination compounds based on the dibazol ligand with transition metals (Co, Ni, Zn and Cu) and studied their physicochemical properties (Babayeva *et al.*, 2025 ▸). New coordination compounds with Fe^II^ and Cu^II^ and the dibazol ligand have been synthesized and their structural and spectroscopic characteristics investigated and described (Imomov *et al.*, 2008 ▸). In the work by Radjabov *et al.* (2016 ▸), the synthesis and physicochemical (structural and spectroscopic) characterization of Zn^II^ coordination compounds with the dibazol ligand are presented, and the structures of the complexes and potential biological activity, are discussed. Lu *et al.* (2003 ▸) report the synthesis and single-crystal structural characterization of coordination complexes containing benzimidazole-based N-donor ligands. The work provides detailed information on the metal coordination environment and supra­molecular packing features relevant for comparison with similar dibazole-based systems. Liu *et al.* (2014 ▸) describe the synthesis and X-ray structures of metal complexes assembled from bis­(benzimidazole) ligands, forming well-defined supra­molecular architectures. The study highlights coordination geometry and inter­molecular contacts, offering structural parallels useful for discussing related dibazole complexes.
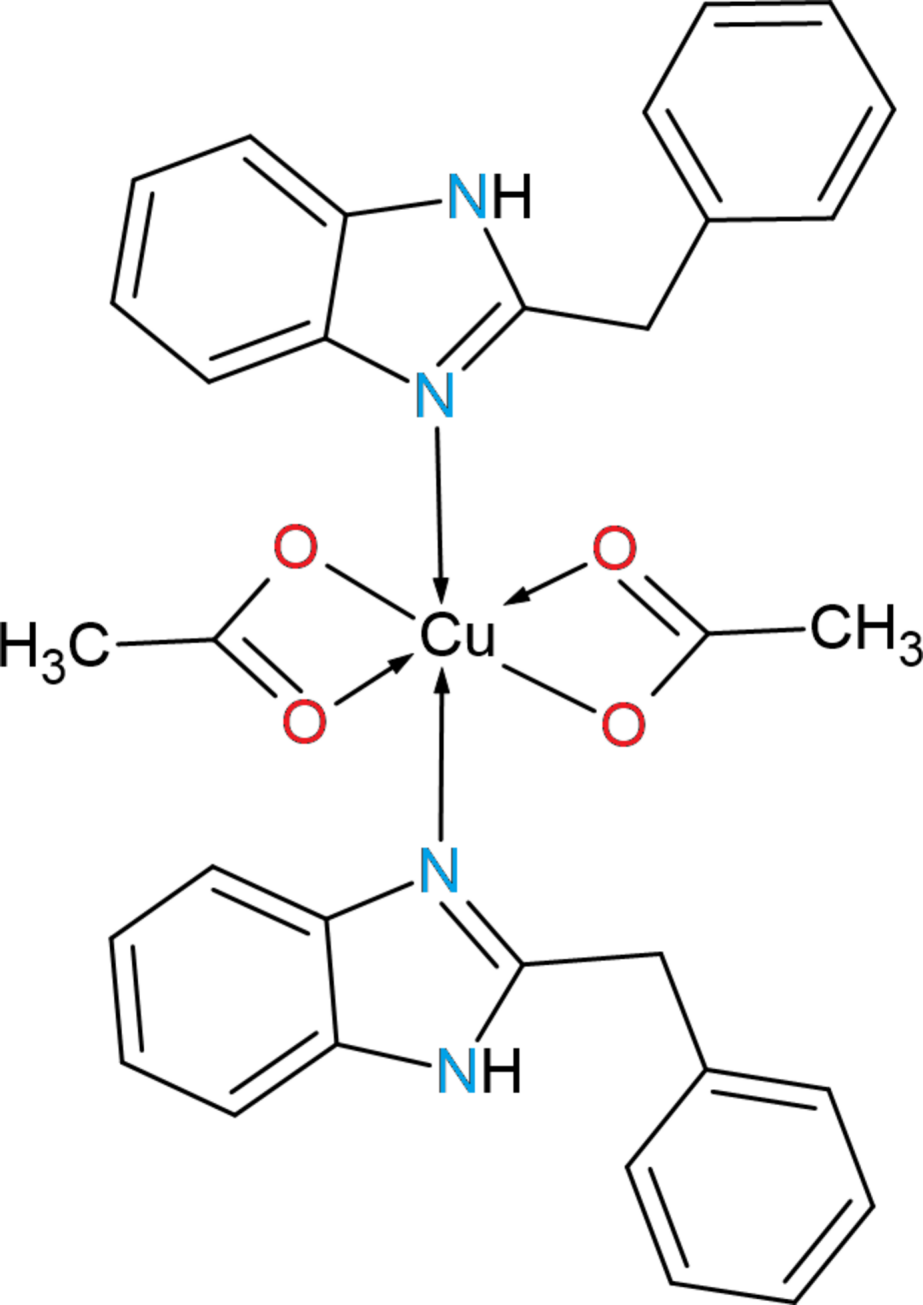


In this connection, we synthesized the title copper(II) complex (**I**). The present work provides an analysis of its structural and supra­molecular properties, Hirshfeld surfaces and DFT calculation analysis.

## 2. Structural commentary

**Figure 1:**
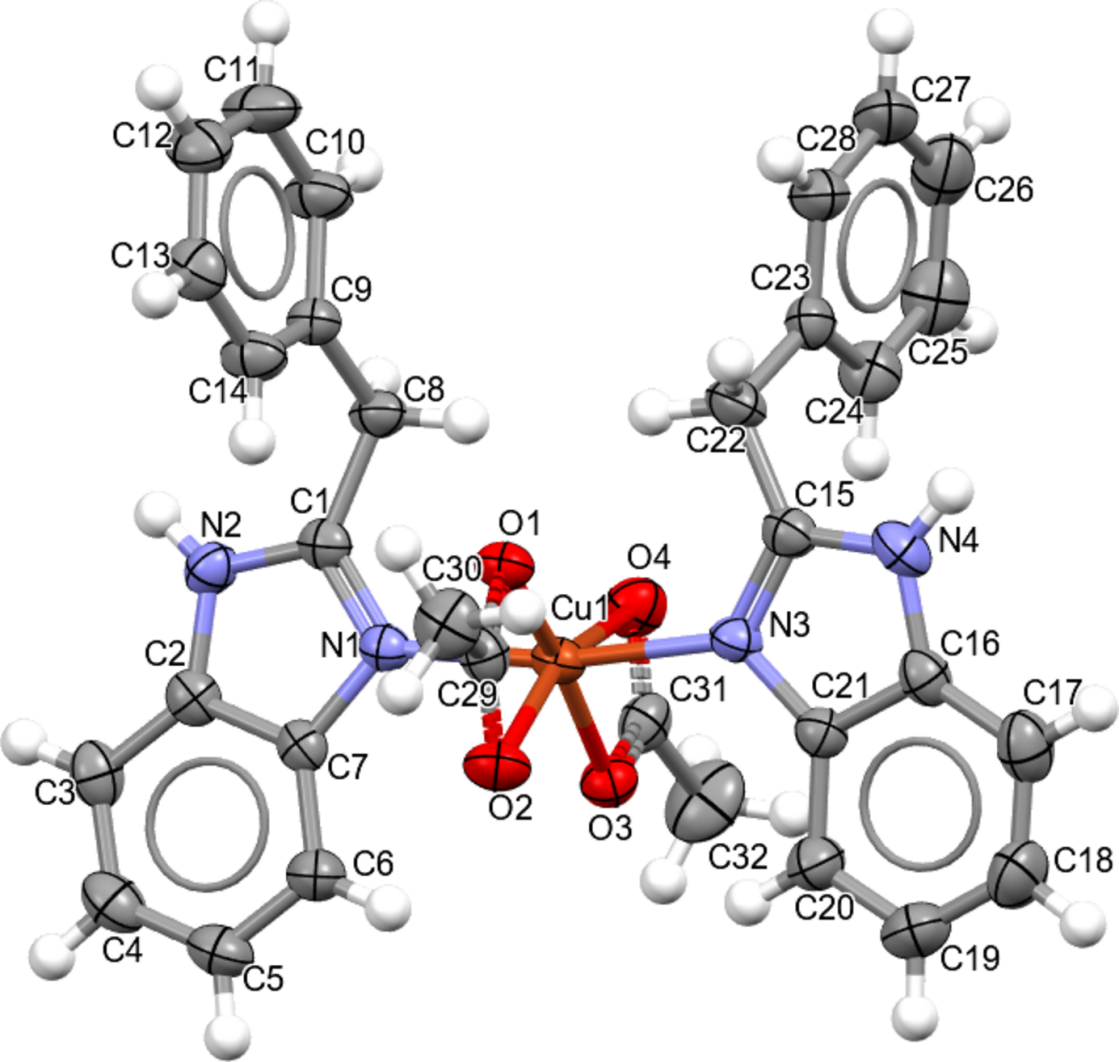
The asymmetric unit of the title compound with the atom-numbering scheme. Displacement ellipsoids for non-hydrogen atoms are drawn at the 50% probability level.

The title compound **I** crystallizes in the monoclinic space group *P2*_1_*/n* (Fig. 1 ▸). The unit cell contains one complex mol­ecule (*Z*′ = 1) in which the central Cu^2+^ ion is coordinated by two mol­ecules of dibazol (DIB) *via sp*^2^-hybridized nitro­gen atoms [Cu—N1 = 1.984 (3) Å and Cu—N3 = 1.986 (3) Å] and two acetate (ac) anions *via* oxygen atoms [Cu—O1 = 1.998 (2) Å, Cu—O2 = 2.447 (2) Å, Cu—O3 = 1.955 (2) Å and Cu—O4 = 2.706 (3) Å]. The acetate ligands form a four-membered chelate ring, in which the chelate angles are O2—Cu—O1 = 57.85 (9)° and O4—Cu—O3 = 53.32 (9)° (Table 1 ▸).

The coordination geometry of the central metal is a distorted octa­hedron (4 + 2). This is explained by the fact that the DIB ligands occupy the axial positions with N1—Cu—N3 = 168.64 (11)° (which deviates by 11.36° from the ideal), as well as by the chelate angles of the ac ligands, which differ significantly from the ideal 90° (Table 1 ▸). This combination of small chelate angles in a constrained geometry forces the axial ligands to deviate and results in elongation of the second oxygen atoms of the ac ligands, together with the Jahn–Teller effect typical for *d*^9^ Cu^II ^ atoms (Jahn & Teller 1937 ▸). An additional contribution may be ascribed to steric inter­actions of the aromatic fragments, which further enhance the departure of the axial donors from 180°.

For consideration of the bidentate nature of the ac ligands, one may refer to Youngme *et al.* (1998 ▸), where the authors obtained an octa­hedral structure with the Cu^2+^ ion and two bidentate ac ligands [Cu—O2 = 2.4824 (15) Å and Cu—O4 = 2.690 (2) Å], the Cu—O bond lengths being very close to those in our structure. The distortion of the Cu coordination is qu­anti­fied as: Σ (θ_*i*_ − 90°| for 12 *cis*-angles = 157.97°, mean absolute deviation ≃ 13.16°; quadratic elongation λ = 1.0178 and Δ = 0.0178. The small chelate angles of the ac ligands [57.85 (9)° and 53.33 (10)°] and the elongated axial bonds (2.447, 2.706 Å) lead to substantial angular and bond-length distortion.

## 3. Supra­molecular features

**Figure 2:**
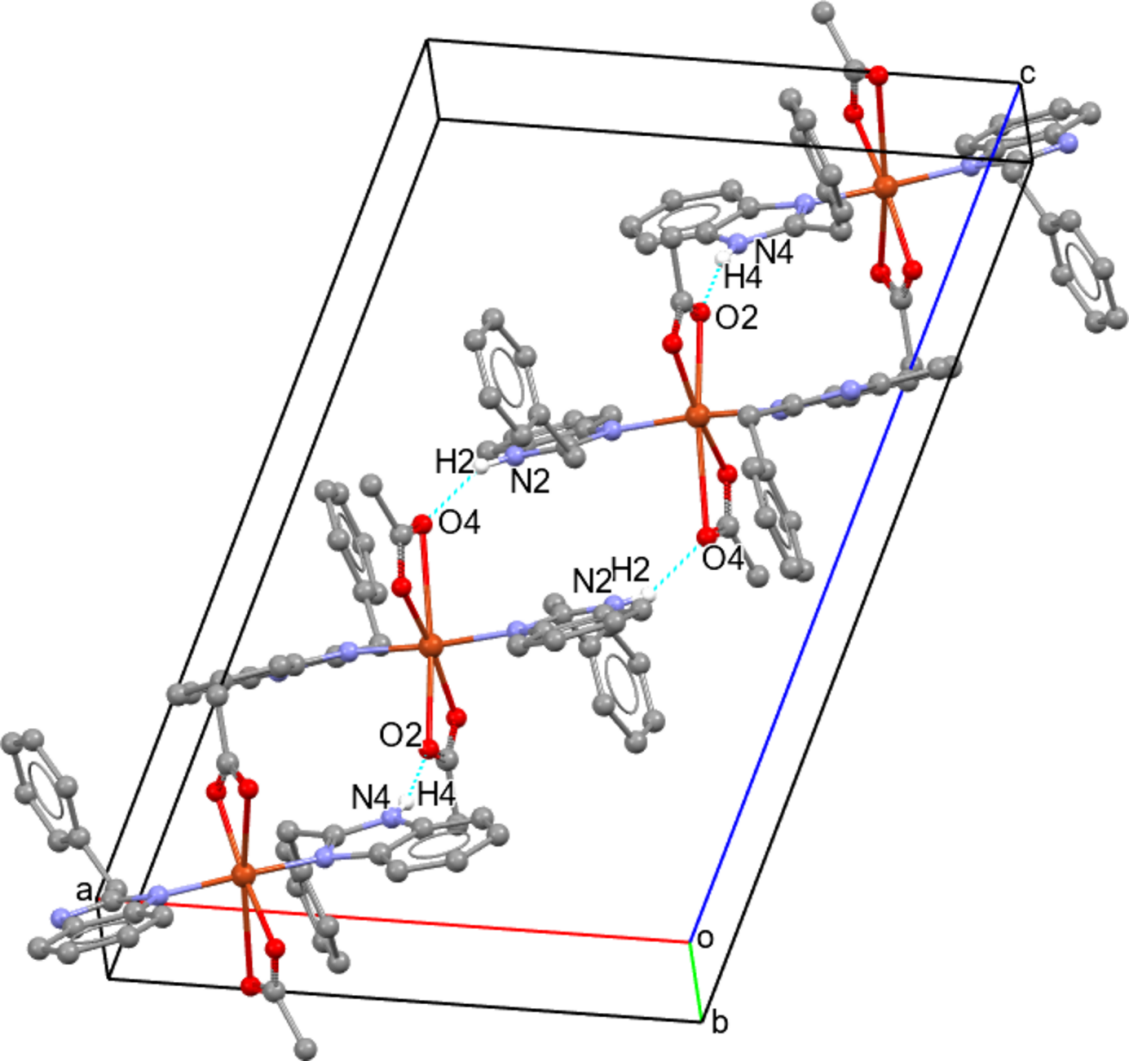
Formation of an inter­molecular chain along [101] by classical N—H⋯O hydrogen bonds. Only hydrogen atoms involved in these inter­actions are shown.

In the crystal, N—H⋯O hydrogen bonds consolidate the structure (Table 2 ▸, Fig. 2 ▸). The formation of the three-dimensional crystal structure is mainly mediated by two principal hydrogen bonds, N2—H2⋯O4^i^ and N4—H4⋯O2^ii^ [symmetry codes: (i) −*x* + 1, −*y* + 1, −*z* + 1; (ii) −*x* + 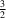, *y* − 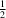, −*z* + 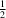] directed along the [101] chain (Table 2 ▸).

**Figure 3:**
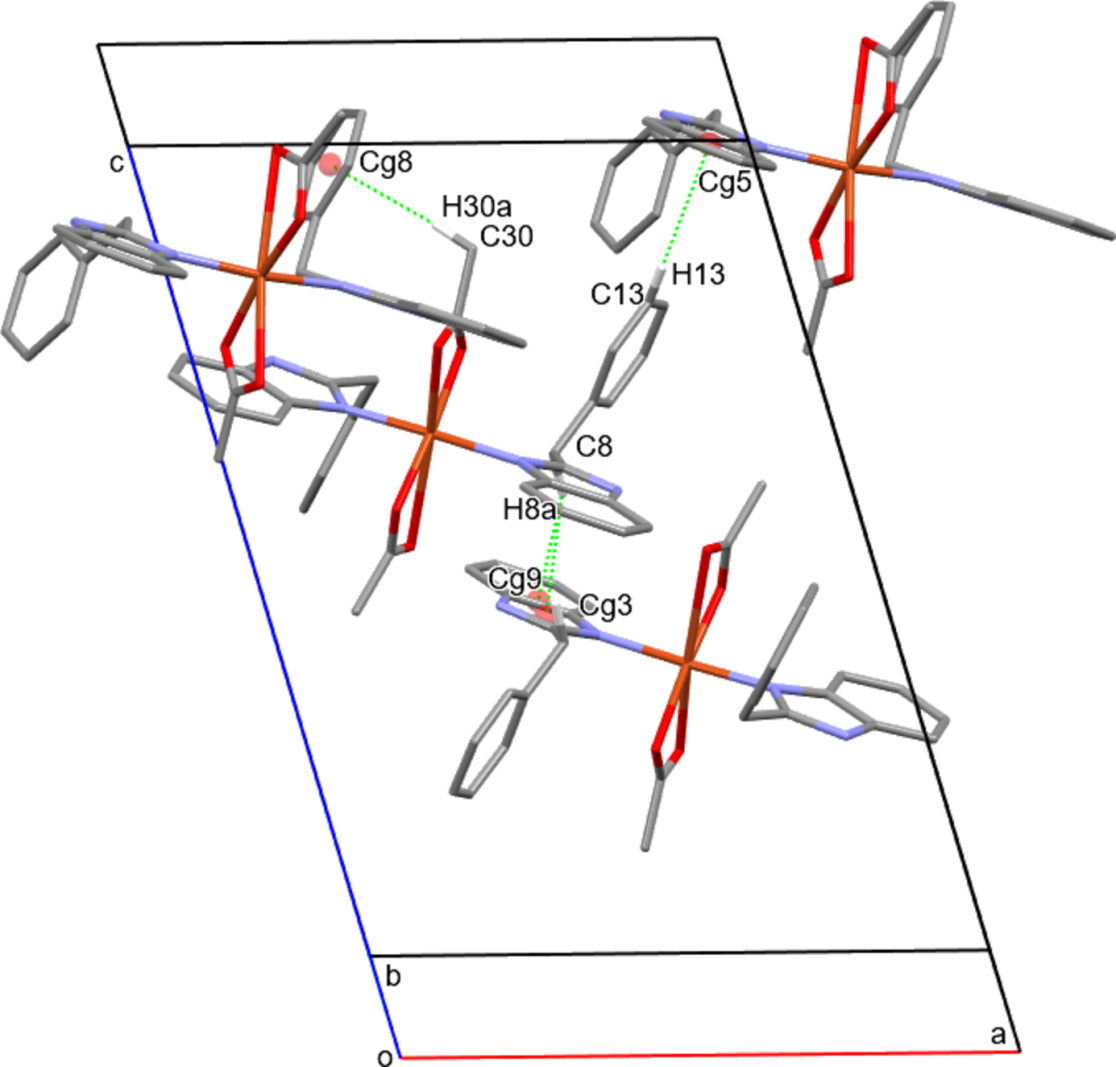
View of the inter­molecular chain along the [011] direction, formed by non-classical hydrogen bonds.

In addition to classical hydrogen bonds, C—H⋯π contacts are present, which further reinforce the crystal cohesion: C8—H8a⋯*Cg*3^i^, C8—H8a⋯*Cg*9^i^, C13—H13⋯*Cg*5^iii^ and C30—H30a⋯*Cg*8^iv^ [(i) −*x* + 1, −*y* + 1, −*z* + 1; (iii) −*x* + 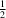, *y* − 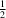, −*z* + 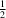; (iv) −*x* + 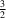, *y* + 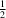, −*z* + 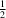] (Table 2 ▸, Fig. 3 ▸). These C—H⋯π inter­actions, although weaker than conventional hydrogen bonds, effect a redistribution of the aromatic fragments within the packing; they promote the orientation of the phenyl systems and stabilize the displaced positions of the rings, which additionally lowers the free energy of the crystal structure.

## 4. Hirshfeld surface analysis

**Figure 4:**
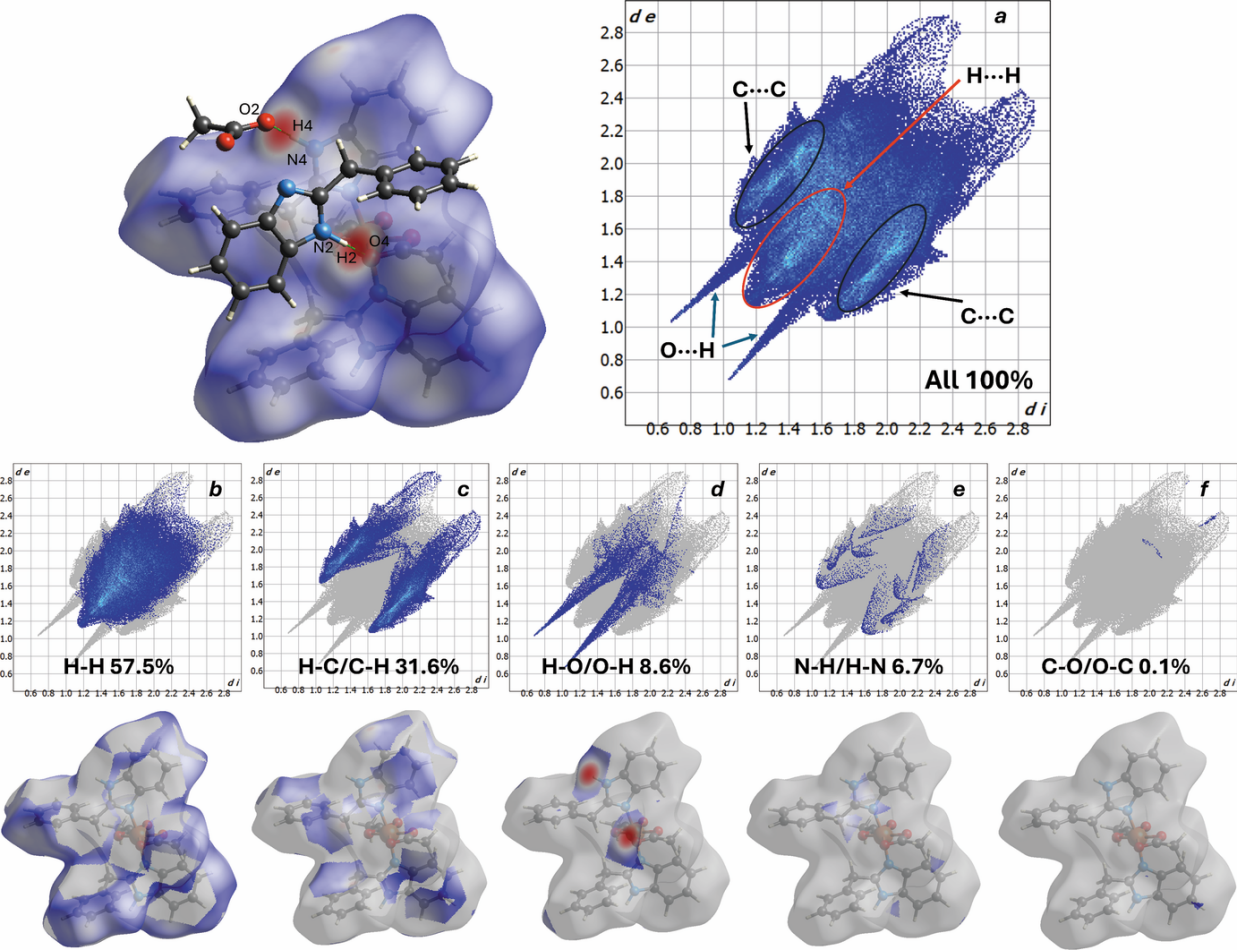
Two-dimensional fingerprint plots for the title compound, showing (*a*) all inter­actions, and delineated into (*b*) H⋯H, (*c*) C⋯H/H⋯C, (*d*) O⋯H/H⋯O, (*e*) N⋯H/H⋯N and (*f*) C⋯O/O⋯C inter­actions.

The Hirshfeld surface analysis was performed using *CrystalExplorer 21.5* (Spackman *et al.*, 2021 ▸). In the *d*_norm_ map (Fig. 4 ▸) the localized dark-red spots correspond to contacts shorter than the sum of the van der Waals radii (close contacts), white areas indicate contacts close to the sum of the radii, and blue areas indicate longer contacts. In the mol­ecule under consideration the most pronounced red regions are observed close to atoms O2/O4 and in the regions between the aromatic rings, which points to the presence of short O⋯H/N or *π–π* contacts in these fragments. Small red spots are also visible on the surface in regions corresponding to the N and H donor atoms, which is consistent with the directional N—H⋯O hydrogen contacts registered in the crystal.

Two-dimensional fingerprint plots (Fig. 4 ▸) provide a qu­anti­tative representation of the contribution of different types of inter­molecular contacts to the total Hirshfeld surface. For the present structure the following proportions are observed: H⋯H = 57.5%, which is the dominant component; H⋯C/C⋯H = 31.6%, which make significant contributions; and the remainder are O⋯H/H⋯O = 8.6%, O⋯C/C⋯O = 6.7% and C⋯O/O⋯C = 0.1%.

The dominance of H⋯H contacts may indicate a predominance of dispersion (van der Waals) contacts and a large number of H–H geometries. The substantial H⋯C/C⋯H contribution reflects edge contacts between aromatic fragments (C—H⋯π), while O⋯H/H⋯O corroborates the presence of directional N—H⋯O and local C—H⋯O inter­actions. For comparison with related complex systems, H⋯H ≃ 71.7% in one case (Siddikova *et al.*, 2024 ▸), whereas in another it is ≃ 51.8% with O⋯H ≃ 12.4% (Tojiboyeva *et al.*, 2025 ▸), highlighting the variability in the balance between dispersion and directional contacts in such structures.

## 5. Database survey

A search of the Cambridge Structural Database (CSD, 2024.2.0; Groom *et al.*, 2016 ▸) returned 52 structures similar to the fragment of our structure. Among these structures a similar zinc complex was identified, in which the central metal resides in a tetra­hedral environment and two dibazol ligands are present (CSD refcode WOVQED; Bei *et al.*, 2001 ▸). The 1,2-phenyl­ene[bis­(methyl­ene)]bis­(1*H*-benzimidazole) ligand with various metals is also frequently encountered [CSD refcodes FUDZEL (Liu *et al.*, 2014 ▸); HUGZUH (Ohta *et al.*, 2020 ▸) and LADLOS (Lu *et al.*, 2003 ▸)].

## 6. Synthesis and crystallization

The following solutions were prepared: (*a*) an ethano­lic solution of Cu(CH_3_COO)_2_·4H_2_O (1.0 mmol) and (*b*) an ethano­lic solution of DIB (2.0 mmol). Solution (*a*) was added to solution (*b*), and the mixture was stirred with a magnetic stirrer at room temperature for 12 h, resulting in the formation of a dark-blue precipitate. The precipitate was filtered, washed several times with ethanol and air-dried. As the obtained material dissolved well in DMF, it was recrystallized from this solvent by dissolution in a minimal volume of DMF followed by slow evaporation; as a result, well-formed single crystals of dark-blue colour, suitable for structural and further physicochemical investigation, were obtained.
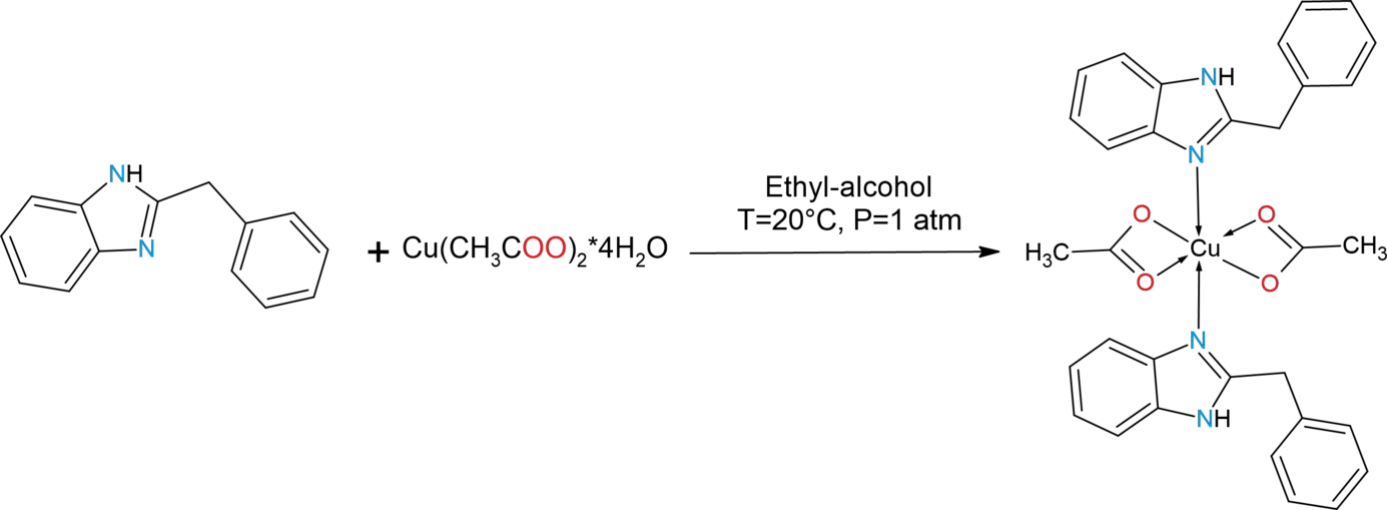


## 7. Refinement

Crystallographic data, data-collection conditions and structure-refinement parameters are summarized in Table 3 ▸. Hydrogen atoms were calculated in idealized positions and refined using a riding model with C—H bond lengths of 0.93–0.98 Å and *U*_iso_(H) = 1.2*U*_eq_(C).

## Supplementary Material

Crystal structure: contains datablock(s) I. DOI: 10.1107/S2056989025010813/ny2018sup1.cif

CCDC reference: 2512906

Additional supporting information:  crystallographic information; 3D view; checkCIF report

## Figures and Tables

**Table 1:** Selected geometric parameters (Å, °)

Cu1—O3	1.955 (2)	Cu1—O4	2.706 (3)
Cu1—O1	1.998 (2)	Cu1—N3	1.986 (3)
Cu1—O2	2.447 (2)	Cu1—N1	1.984 (3)
			
O2—Cu1—O1	57.85 (9)	N1—Cu1—N3	168.64 (11)
O4—Cu1—O3	53.32 (9)		

**Table 2:** Hydrogen-bond geometry (Å, °) *Cg*3, 5, 8 and 9 are the centroids of the N1/C1/N2/C2/C7, C2–C7, C23–C28 and N1/C1/N2/C2–C7 rings, respectively

*D*—H⋯*A*	*D*—H	H⋯*A*	*D*⋯*A*	*D*—H⋯*A*
N2—H2⋯O4^i^	0.86 (1)	1.91 (1)	2.708 (4)	153 (1)
N4—H4⋯O2^ii^	0.86 (1)	1.86 (1)	2.699 (4)	164 (1)
C8—H8*a*⋯*Cg*3^i^	0.97 (1)	2.70 (1)	3.592 (4)	154 (1)
C8—H8*a*⋯*Cg*9^i^	0.97 (1)	2.93 (1)	3.844 (4)	158 (1)
C13—H13⋯*Cg*5^iii^	0.93 (1)	2.82 (1)	3.614 (4)	145 (1)
C30—H30*a*⋯*Cg*8^iv^	0.96 (1)	2.93 (1)	3.690 (5)	137 (2)

Symmetry codes: (i) 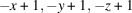; (ii) 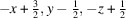; (iii) 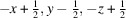; (iv) 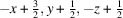.

**Table 3:** Experimental details

Crystal data	
Chemical formula	[Cu(C_2_H_3_O_2_)_2_(C_14_H_12_N_2_)_2_]
*M*_r_	598.17
Crystal system, space group	Monoclinic, *P*2_1_/*n*
Temperature (K)	273
*a*, *b*, *c* (Å)	13.125 (4), 11.552 (3), 20.499 (5)
β (°)	106.385 (12)
*V* (Å^3^)	2981.9 (13)
*Z*	4
Radiation type	Mo *K*α
μ (mm^−1^)	0.78
Crystal size (mm)	0.45 × 0.35 × 0.18

Data collection	
Diffractometer	Bruker APEXII CCD
Absorption correction	Multi-scan (*SADABS*; Krause *et al.*, 2015 ▸)
*T*_min_, *T*_max_	0.672, 0.754
No. of measured, independent and observed [*I* ≥ 2u(*I*)] reflections	7459, 7455, 3700
*R*_int_	0.055
(sin θ/λ)_max_ (Å^−1^)	0.670

Refinement	
*R*[*F*^2^ > 2σ(*F*^2^)], *wR*(*F*^2^), *S*	0.069, 0.137, 0.98
No. of reflections	7455
No. of parameters	372
H-atom treatment	H atoms treated by a mixture of independent and constrained refinement
Δρ_max_, Δρ_min_ (e Å^−3^)	0.22, −1.15

Computer programs: *APEX2* and *SAINT* (Bruker, 2014 ▸), *OLEX2.refine* (Bourhis *et al.*, 2015 ▸), *OLEX2* (Dolomanov *et al.*, 2009 ▸) and *Mercury*(Macrae *et al.*, 2020 ▸) .

## supplementary crystallographic information

### Bis(acetato-κ2O,O')(2-benzyl-1H-benzimidazole-κN3)copper(II) Crystal data

**Table:**

[Cu(C_2_H_3_O_2_)_2_(C_14_H_12_N_2_)_2_]	*F*(000) = 1244
*M**_r_* = 598.17	*D*_x_ = 1.332 Mg m^−^^3^
Monoclinic, *P*2_1_/*n*	Mo *K*α radiation, λ = 0.71073 Å
*a* = 13.125 (4) Å	Cell parameters from 9790 reflections
*b* = 11.552 (3) Å	θ = 2.2–22.5°
*c* = 20.499 (5) Å	µ = 0.78 mm^−^^1^
β = 106.385 (12)°	*T* = 273 K
*V* = 2981.9 (13) Å^3^	Rhombohedral, clear dark blue
*Z* = 4	0.45 × 0.35 × 0.18 mm

### Bis(acetato-κ2O,O')(2-benzyl-1H-benzimidazole-κN3)copper(II) Data collection

**Table:**

Bruker APEXII CCD diffractometer	3700 reflections with *I*≥ 2u(*I*)
ω scans	*R*_int_ = 0.055
Absorption correction: multi-scan (SADABS; Krause *et al.*, 2015)	θ_max_ = 28.4°, θ_min_ = 2.1°
*T*_min_ = 0.672, *T*_max_ = 0.754	*h* = −17→16
7459 measured reflections	*k* = 0→15
7455 independent reflections	*l* = 0→27

### Bis(acetato-κ2O,O')(2-benzyl-1H-benzimidazole-κN3)copper(II) Refinement

**Table:**

Refinement on *F*^2^	54 constraints
Least-squares matrix: full	Primary atom site location: dual
*R*[*F*^2^ > 2σ(*F*^2^)] = 0.069	Hydrogen site location: inferred from neighbouring sites
*wR*(*F*^2^) = 0.137	H atoms treated by a mixture of independent and constrained refinement
*S* = 0.98	*w* = 1/[σ^2^(*F*_o_^2^) + (0.0369*P*)^2^ + 1.6134*P*] where *P* = (*F*_o_^2^ + 2*F*_c_^2^)/3
7455 reflections	(Δ/σ)_max_ = −0.001
372 parameters	Δρ_max_ = 0.22 e Å^−^^3^
0 restraints	Δρ_min_ = −1.15 e Å^−^^3^

### Bis(acetato-κ2O,O')(2-benzyl-1H-benzimidazole-κN3)copper(II) Fractional atomic coordinates and isotropic or equivalent isotropic displacement parameters (Å^2^)

**Table:**

	*x*	*y*	*z*	*U*_iso_*/*U*_eq_	
Cu1	0.65105 (3)	0.56975 (3)	0.36489 (2)	0.05030 (16)	
O3	0.74289 (19)	0.6635 (2)	0.43733 (12)	0.0590 (6)	
O1	0.55906 (19)	0.49963 (19)	0.27936 (11)	0.0567 (6)	
O2	0.5973 (2)	0.6771 (2)	0.25771 (12)	0.0683 (7)	
O4	0.7374 (2)	0.5054 (3)	0.49579 (13)	0.0756 (8)	
N3	0.7774 (2)	0.5000 (2)	0.34504 (13)	0.0510 (7)	
N1	0.5217 (2)	0.6087 (2)	0.39222 (13)	0.0486 (7)	
N4	0.8712 (3)	0.3728 (3)	0.30583 (15)	0.0687 (9)	
H4	0.8904 (3)	0.3075 (3)	0.29297 (15)	0.0824 (11)*	
N2	0.3780 (2)	0.5905 (3)	0.42757 (14)	0.0580 (8)	
H2	0.3265 (2)	0.5582 (3)	0.43903 (14)	0.0696 (9)*	
C9	0.3743 (3)	0.3421 (3)	0.35704 (17)	0.0506 (9)	
C21	0.8626 (3)	0.5572 (3)	0.33110 (17)	0.0529 (9)	
C7	0.4857 (3)	0.7190 (3)	0.40332 (16)	0.0491 (9)	
C16	0.9217 (3)	0.4783 (3)	0.30590 (18)	0.0596 (10)	
C2	0.3965 (3)	0.7082 (3)	0.42631 (16)	0.0538 (9)	
C31	0.7724 (3)	0.6038 (4)	0.4909 (2)	0.0620 (10)	
C23	0.7691 (3)	0.2023 (3)	0.3881 (2)	0.0592 (10)	
C15	0.7871 (3)	0.3910 (3)	0.32937 (18)	0.0569 (10)	
C29	0.5540 (3)	0.5827 (3)	0.23876 (18)	0.0560 (9)	
C6	0.5265 (3)	0.8277 (3)	0.39576 (19)	0.0640 (10)	
H6	0.5856 (3)	0.8360 (3)	0.37959 (19)	0.0767 (13)*	
C1	0.4540 (3)	0.5358 (3)	0.40787 (17)	0.0519 (9)	
C3	0.3461 (3)	0.8038 (4)	0.44419 (18)	0.0690 (11)	
H3	0.2865 (3)	0.7964 (4)	0.45993 (18)	0.0829 (13)*	
C10	0.3509 (3)	0.2312 (3)	0.3720 (2)	0.0766 (12)	
H10	0.3886 (3)	0.1972 (3)	0.4127 (2)	0.0919 (15)*	
C4	0.3893 (4)	0.9103 (4)	0.4373 (2)	0.0777 (12)	
H4a	0.3585 (4)	0.9765 (4)	0.4494 (2)	0.0933 (15)*	
C5	0.4766 (4)	0.9219 (3)	0.4130 (2)	0.0745 (12)	
H5	0.5022 (4)	0.9956 (3)	0.4082 (2)	0.0893 (14)*	
C24	0.8457 (3)	0.2304 (4)	0.4472 (2)	0.0739 (12)	
H24	0.8666 (3)	0.3072 (4)	0.4557 (2)	0.0887 (14)*	
C20	0.8934 (3)	0.6734 (3)	0.3386 (2)	0.0713 (11)	
H20	0.8556 (3)	0.7278 (3)	0.3558 (2)	0.0855 (14)*	
C28	0.7402 (3)	0.0879 (3)	0.3766 (2)	0.0762 (12)	
H28	0.6892 (3)	0.0671 (3)	0.3367 (2)	0.0915 (14)*	
C12	0.2139 (3)	0.2187 (4)	0.2669 (2)	0.0797 (13)	
H12	0.1589 (3)	0.1779 (4)	0.2373 (2)	0.0957 (15)*	
C13	0.2376 (3)	0.3269 (4)	0.2514 (2)	0.0722 (11)	
H13	0.1998 (3)	0.3604 (4)	0.2104 (2)	0.0866 (14)*	
C14	0.3181 (3)	0.3884 (3)	0.29639 (19)	0.0686 (11)	
H14	0.3342 (3)	0.4628 (3)	0.28497 (19)	0.0823 (13)*	
C11	0.2710 (4)	0.1696 (4)	0.3262 (3)	0.0934 (15)	
H11	0.2565 (4)	0.0940 (4)	0.3363 (3)	0.1121 (18)*	
C8	0.4632 (3)	0.4065 (3)	0.40743 (19)	0.0601 (10)	
H8a	0.4675 (3)	0.3783 (3)	0.45269 (19)	0.0722 (12)*	
H8b	0.5296 (3)	0.3870 (3)	0.39805 (19)	0.0722 (12)*	
C30	0.4952 (3)	0.5636 (4)	0.16491 (18)	0.0806 (13)	
H30a	0.5427 (7)	0.531 (2)	0.1418 (4)	0.1210 (19)*	
H30b	0.4370 (14)	0.5115 (19)	0.16164 (19)	0.1210 (19)*	
H30c	0.4687 (19)	0.6362 (5)	0.1443 (4)	0.1210 (19)*	
C17	1.0109 (4)	0.5101 (5)	0.2864 (2)	0.0842 (13)	
H17	1.0495 (4)	0.4564 (5)	0.2693 (2)	0.1010 (16)*	
C27	0.7867 (4)	0.0026 (4)	0.4239 (3)	0.0944 (15)	
H27	0.7665 (4)	−0.0745 (4)	0.4163 (3)	0.1133 (18)*	
C26	0.8620 (4)	0.0346 (5)	0.4815 (3)	0.0995 (16)	
H26	0.8938 (4)	−0.0216 (5)	0.5132 (3)	0.1194 (19)*	
C19	0.9819 (4)	0.7035 (4)	0.3195 (2)	0.0898 (14)	
H19	1.0043 (4)	0.7802 (4)	0.3240 (2)	0.1077 (17)*	
C22	0.7159 (3)	0.2936 (3)	0.3370 (2)	0.0750 (12)	
H22a	0.6876 (3)	0.2568 (3)	0.2930 (2)	0.0900 (15)*	
H22b	0.6565 (3)	0.3255 (3)	0.3504 (2)	0.0900 (15)*	
C25	0.8914 (4)	0.1469 (5)	0.4937 (2)	0.0930 (15)	
H25	0.9427 (4)	0.1671 (5)	0.5335 (2)	0.1116 (17)*	
C18	1.0388 (4)	0.6243 (5)	0.2937 (3)	0.0949 (15)	
H18	1.0978 (4)	0.6492 (5)	0.2809 (3)	0.1139 (18)*	
C32	0.8565 (4)	0.6567 (5)	0.5493 (2)	0.1121 (18)	
H32a	0.9253 (4)	0.643 (3)	0.5431 (10)	0.168 (3)*	
H32b	0.8446 (17)	0.7385 (7)	0.5507 (11)	0.168 (3)*	
H32c	0.8530 (19)	0.622 (2)	0.5912 (3)	0.168 (3)*	

### Bis(acetato-κ2O,O')(2-benzyl-1H-benzimidazole-κN3)copper(II) Atomic displacement parameters (Å^2^)

**Table:**

	*U*^11^	*U*^22^	*U*^33^	*U*^12^	*U*^13^	*U*^23^
Cu1	0.0582 (3)	0.0423 (2)	0.0496 (3)	−0.0014 (2)	0.0138 (2)	−0.0012 (2)
O3	0.0619 (17)	0.0580 (15)	0.0562 (16)	−0.0054 (13)	0.0153 (13)	−0.0042 (13)
O1	0.0690 (17)	0.0448 (14)	0.0542 (14)	−0.0030 (12)	0.0142 (13)	0.0026 (12)
O2	0.087 (2)	0.0511 (16)	0.0672 (17)	−0.0064 (14)	0.0220 (15)	0.0064 (13)
O4	0.082 (2)	0.079 (2)	0.0721 (18)	−0.0047 (16)	0.0324 (16)	0.0088 (16)
N3	0.059 (2)	0.0401 (16)	0.0551 (18)	−0.0007 (14)	0.0181 (15)	−0.0049 (14)
N1	0.0528 (18)	0.0447 (16)	0.0470 (16)	−0.0018 (14)	0.0117 (14)	−0.0011 (13)
N4	0.082 (2)	0.053 (2)	0.074 (2)	0.0147 (18)	0.026 (2)	−0.0052 (16)
N2	0.055 (2)	0.062 (2)	0.0597 (19)	−0.0049 (16)	0.0206 (16)	0.0063 (15)
C9	0.056 (2)	0.044 (2)	0.052 (2)	−0.0024 (17)	0.0149 (19)	0.0013 (17)
C21	0.057 (2)	0.051 (2)	0.051 (2)	0.0005 (19)	0.0160 (18)	−0.0001 (17)
C7	0.052 (2)	0.051 (2)	0.0410 (19)	0.0049 (18)	0.0078 (18)	−0.0023 (16)
C16	0.066 (3)	0.061 (2)	0.054 (2)	0.011 (2)	0.021 (2)	0.0021 (19)
C2	0.063 (3)	0.059 (2)	0.0395 (19)	0.005 (2)	0.0148 (19)	0.0004 (17)
C31	0.057 (3)	0.078 (3)	0.056 (2)	−0.006 (2)	0.024 (2)	−0.008 (2)
C23	0.057 (2)	0.057 (2)	0.069 (3)	0.0044 (19)	0.026 (2)	0.000 (2)
C15	0.061 (3)	0.050 (2)	0.057 (2)	0.0023 (19)	0.011 (2)	0.0013 (18)
C29	0.057 (2)	0.062 (3)	0.052 (2)	0.008 (2)	0.0193 (19)	0.001 (2)
C6	0.067 (3)	0.053 (2)	0.073 (3)	−0.004 (2)	0.020 (2)	−0.001 (2)
C1	0.055 (2)	0.051 (2)	0.044 (2)	−0.0006 (19)	0.0046 (18)	0.0027 (16)
C3	0.072 (3)	0.083 (3)	0.056 (2)	0.016 (2)	0.024 (2)	−0.001 (2)
C10	0.083 (3)	0.055 (2)	0.079 (3)	−0.008 (2)	0.001 (2)	0.015 (2)
C4	0.098 (4)	0.064 (3)	0.070 (3)	0.014 (3)	0.021 (3)	−0.010 (2)
C5	0.092 (3)	0.049 (2)	0.080 (3)	−0.001 (2)	0.021 (3)	−0.005 (2)
C24	0.076 (3)	0.081 (3)	0.064 (3)	−0.002 (2)	0.019 (2)	−0.008 (2)
C20	0.068 (3)	0.065 (3)	0.084 (3)	−0.008 (2)	0.027 (2)	−0.007 (2)
C28	0.070 (3)	0.056 (3)	0.097 (3)	−0.007 (2)	0.015 (2)	0.004 (2)
C12	0.079 (3)	0.064 (3)	0.085 (3)	−0.009 (2)	0.005 (3)	−0.011 (2)
C13	0.076 (3)	0.074 (3)	0.057 (2)	0.004 (2)	0.003 (2)	0.000 (2)
C14	0.080 (3)	0.055 (2)	0.062 (3)	−0.007 (2)	0.007 (2)	0.010 (2)
C11	0.107 (4)	0.054 (3)	0.106 (4)	−0.023 (3)	0.008 (3)	0.006 (3)
C8	0.063 (3)	0.046 (2)	0.066 (2)	−0.0074 (18)	0.010 (2)	0.0080 (18)
C30	0.084 (3)	0.100 (3)	0.053 (2)	−0.001 (3)	0.011 (2)	0.004 (2)
C17	0.081 (3)	0.099 (4)	0.082 (3)	0.020 (3)	0.038 (3)	0.011 (3)
C27	0.081 (3)	0.066 (3)	0.140 (5)	−0.003 (3)	0.037 (4)	0.029 (3)
C26	0.076 (4)	0.117 (5)	0.107 (4)	0.009 (3)	0.029 (3)	0.044 (3)
C19	0.089 (4)	0.072 (3)	0.113 (4)	−0.017 (3)	0.035 (3)	0.004 (3)
C22	0.078 (3)	0.046 (2)	0.093 (3)	0.004 (2)	0.013 (3)	0.004 (2)
C25	0.090 (4)	0.123 (4)	0.067 (3)	0.005 (3)	0.024 (3)	0.005 (3)
C18	0.079 (4)	0.103 (4)	0.117 (4)	−0.003 (3)	0.049 (3)	0.009 (3)
C32	0.100 (4)	0.154 (5)	0.069 (3)	−0.027 (4)	0.001 (3)	−0.019 (3)

### Bis(acetato-κ2O,O')(2-benzyl-1H-benzimidazole-κN3)copper(II) Geometric parameters (Å, º)

**Table:**

Cu1—O3	1.955 (2)	C3—C4	1.378 (5)
Cu1—O1	1.998 (2)	C10—H10	0.9300
Cu1—O2	2.447 (2)	C10—C11	1.389 (5)
Cu1—O4	2.706 (3)	C4—H4a	0.9300
Cu1—N3	1.986 (3)	C4—C5	1.380 (6)
Cu1—N1	1.984 (3)	C5—H5	0.9300
O3—C31	1.261 (4)	C24—H24	0.9300
O1—C29	1.260 (4)	C24—C25	1.370 (6)
O2—C29	1.240 (4)	C20—H20	0.9300
O4—C31	1.240 (4)	C20—C19	1.372 (5)
N3—C21	1.395 (4)	C28—H28	0.9300
N3—C15	1.315 (4)	C28—C27	1.396 (6)
N1—C7	1.399 (4)	C12—H12	0.9300
N1—C1	1.328 (4)	C12—C13	1.348 (5)
N4—H4	0.8600	C12—C11	1.359 (6)
N4—C16	1.388 (5)	C13—H13	0.9300
N4—C15	1.340 (4)	C13—C14	1.387 (5)
N2—H2	0.8600	C14—H14	0.9300
N2—C2	1.383 (4)	C11—H11	0.9300
N2—C1	1.336 (4)	C8—H8a	0.9700
C9—C10	1.371 (5)	C8—H8b	0.9700
C9—C14	1.363 (5)	C30—H30a	0.9600
C9—C8	1.518 (4)	C30—H30b	0.9600
C21—C16	1.388 (5)	C30—H30c	0.9600
C21—C20	1.397 (5)	C17—H17	0.9300
C7—C2	1.386 (5)	C17—C18	1.367 (6)
C7—C6	1.392 (5)	C27—H27	0.9300
C16—C17	1.388 (5)	C27—C26	1.360 (6)
C2—C3	1.389 (5)	C26—H26	0.9300
C31—C32	1.510 (5)	C26—C25	1.356 (7)
C23—C24	1.379 (5)	C19—H19	0.9300
C23—C28	1.377 (5)	C19—C18	1.377 (6)
C23—C22	1.511 (5)	C22—H22a	0.9700
C15—C22	1.498 (5)	C22—H22b	0.9700
C29—C30	1.509 (5)	C25—H25	0.9300
C6—H6	0.9300	C18—H18	0.9300
C6—C5	1.366 (5)	C32—H32a	0.9600
C1—C8	1.499 (4)	C32—H32b	0.9600
C3—H3	0.9300	C32—H32c	0.9600
			
Cg1···Cg3	3.649 (2)	Cg2···Cg3	3.564 (2)
Cg1···Cg4	3.497 (2)	Cg2···Cg4	3.629 (2)
			
O1—Cu1—O3	168.98 (10)	H4a—C4—C3	119.0 (3)
O2—Cu1—O3	111.18 (10)	C5—C4—C3	122.1 (4)
O2—Cu1—O1	57.85 (9)	C5—C4—H4a	119.0 (2)
O4—Cu1—O3	53.32 (9)	C4—C5—C6	121.6 (4)
O4—Cu1—O1	137.66 (9)	H5—C5—C6	119.2 (3)
O4—Cu1—O2	164.49 (9)	H5—C5—C4	119.2 (2)
N3—Cu1—O3	90.49 (10)	H24—C24—C23	119.5 (2)
N3—Cu1—O1	89.35 (10)	C25—C24—C23	120.9 (4)
N3—Cu1—O2	93.85 (10)	C25—C24—H24	119.5 (3)
N3—Cu1—O4	87.22 (10)	H20—C20—C21	121.5 (2)
N1—Cu1—O3	93.04 (10)	C19—C20—C21	117.0 (4)
N1—Cu1—O1	89.23 (10)	C19—C20—H20	121.5 (3)
N1—Cu1—O2	94.92 (10)	H28—C28—C23	119.6 (2)
N1—Cu1—O4	86.25 (9)	C27—C28—C23	120.9 (4)
N1—Cu1—N3	168.64 (11)	C27—C28—H28	119.6 (3)
C31—O3—Cu1	109.3 (2)	C13—C12—H12	120.2 (3)
C29—O1—Cu1	100.2 (2)	C11—C12—H12	120.2 (3)
C29—O2—Cu1	80.0 (2)	C11—C12—C13	119.5 (4)
C31—O4—Cu1	74.2 (2)	H13—C13—C12	120.0 (3)
C21—N3—Cu1	127.8 (2)	C14—C13—C12	120.1 (4)
C15—N3—Cu1	125.6 (3)	C14—C13—H13	120.0 (2)
C15—N3—C21	105.4 (3)	C13—C14—C9	121.3 (4)
C7—N1—Cu1	127.4 (2)	H14—C14—C9	119.3 (2)
C1—N1—Cu1	127.5 (2)	H14—C14—C13	119.3 (2)
C1—N1—C7	105.0 (3)	C12—C11—C10	120.7 (4)
C16—N4—H4	126.1 (2)	H11—C11—C10	119.6 (2)
C15—N4—H4	126.1 (2)	H11—C11—C12	119.6 (3)
C15—N4—C16	107.7 (3)	C1—C8—C9	116.4 (3)
C2—N2—H2	126.0 (2)	H8a—C8—C9	108.18 (19)
C1—N2—H2	126.0 (2)	H8a—C8—C1	108.2 (2)
C1—N2—C2	108.0 (3)	H8b—C8—C9	108.2 (2)
C14—C9—C10	118.2 (3)	H8b—C8—C1	108.2 (2)
C8—C9—C10	118.9 (3)	H8b—C8—H8a	107.3
C8—C9—C14	122.8 (3)	H30a—C30—C29	109.5
C16—C21—N3	109.3 (3)	H30b—C30—C29	109.5
C20—C21—N3	131.0 (3)	H30b—C30—H30a	109.5
C20—C21—C16	119.8 (4)	H30c—C30—C29	109.5
C2—C7—N1	109.3 (3)	H30c—C30—H30a	109.5
C6—C7—N1	130.2 (4)	H30c—C30—H30b	109.5
C6—C7—C2	120.5 (3)	H17—C17—C16	121.8 (3)
C21—C16—N4	105.0 (3)	C18—C17—C16	116.5 (4)
C17—C16—N4	132.3 (4)	C18—C17—H17	121.8 (3)
C17—C16—C21	122.6 (4)	H27—C27—C28	120.6 (3)
C7—C2—N2	105.3 (3)	C26—C27—C28	118.7 (4)
C3—C2—N2	132.7 (4)	C26—C27—H27	120.6 (3)
C3—C2—C7	122.0 (4)	H26—C26—C27	119.4 (3)
O4—C31—O3	122.8 (4)	C25—C26—C27	121.3 (5)
C32—C31—O3	116.5 (4)	C25—C26—H26	119.4 (3)
C32—C31—O4	120.7 (4)	H19—C19—C20	118.8 (3)
C28—C23—C24	118.3 (4)	C18—C19—C20	122.4 (4)
C22—C23—C24	121.7 (4)	C18—C19—H19	118.8 (3)
C22—C23—C28	120.0 (4)	C15—C22—C23	114.6 (3)
N4—C15—N3	112.6 (3)	H22a—C22—C23	108.6 (2)
C22—C15—N3	126.0 (4)	H22a—C22—C15	108.6 (2)
C22—C15—N4	121.4 (3)	H22b—C22—C23	108.6 (2)
O2—C29—O1	121.9 (3)	H22b—C22—C15	108.6 (2)
C30—C29—O1	117.9 (3)	H22b—C22—H22a	107.6
C30—C29—O2	120.2 (3)	C26—C25—C24	120.0 (5)
H6—C6—C7	121.2 (2)	H25—C25—C24	120.0 (3)
C5—C6—C7	117.6 (4)	H25—C25—C26	120.0 (3)
C5—C6—H6	121.2 (3)	C19—C18—C17	121.7 (5)
N2—C1—N1	112.4 (3)	H18—C18—C17	119.2 (3)
C8—C1—N1	124.8 (3)	H18—C18—C19	119.2 (3)
C8—C1—N2	122.8 (3)	H32a—C32—C31	109.5
H3—C3—C2	121.8 (3)	H32b—C32—C31	109.5
C4—C3—C2	116.3 (4)	H32b—C32—H32a	109.5
C4—C3—H3	121.8 (3)	H32c—C32—C31	109.5
H10—C10—C9	120.0 (2)	H32c—C32—H32a	109.5
C11—C10—C9	120.1 (4)	H32c—C32—H32b	109.5
C11—C10—H10	120.0 (2)		
			
Cu1—O3—C31—O4	−6.6 (2)	C21—C20—C19—C18	0.2 (5)
Cu1—O3—C31—C32	171.4 (3)	C7—N1—C1—C8	−176.6 (2)
Cu1—O1—C29—O2	−2.0 (2)	C7—C2—N2—C1	1.4 (3)
Cu1—O1—C29—C30	176.4 (2)	C7—C2—C3—C4	0.5 (4)
Cu1—O2—C29—O1	1.7 (2)	C7—C6—C5—C4	0.3 (4)
Cu1—O2—C29—C30	−176.75 (19)	C16—N4—C15—C22	−178.9 (3)
Cu1—O4—C31—O3	4.7 (2)	C16—C21—N3—C15	0.8 (3)
Cu1—O4—C31—C32	−173.3 (2)	C16—C21—C20—C19	0.7 (4)
Cu1—N3—C21—C16	−167.2 (3)	C16—C17—C18—C19	0.4 (5)
Cu1—N3—C21—C20	12.8 (4)	C2—N2—C1—C8	176.0 (2)
Cu1—N3—C15—N4	167.8 (3)	C2—C7—N1—C1	0.6 (3)
Cu1—N3—C15—C22	−13.2 (3)	C2—C7—C6—C5	1.2 (4)
Cu1—N1—C7—C2	−175.8 (3)	C2—C3—C4—C5	1.0 (4)
Cu1—N1—C7—C6	3.0 (3)	C23—C24—C25—C26	0.4 (5)
Cu1—N1—C1—N2	176.7 (3)	C23—C28—C27—C26	−0.7 (5)
Cu1—N1—C1—C8	−0.2 (3)	C15—N3—C21—C20	−179.2 (3)
N3—C21—C16—N4	−0.7 (3)	C15—N4—C16—C17	−179.3 (3)
N3—C21—C16—C17	179.0 (3)	C15—C22—C23—C24	36.0 (4)
N3—C21—C20—C19	−179.4 (4)	C15—C22—C23—C28	−144.9 (4)
N3—C15—N4—C16	0.1 (3)	C6—C7—N1—C1	179.4 (4)
N3—C15—C22—C23	−115.1 (4)	C6—C7—C2—C3	−1.6 (4)
N1—C7—C2—N2	−1.2 (3)	C6—C5—C4—C3	−1.5 (5)
N1—C7—C2—C3	177.3 (3)	C1—N2—C2—C3	−176.9 (3)
N1—C7—C6—C5	−177.5 (4)	C1—C8—C9—C10	−151.8 (4)
N1—C1—N2—C2	−1.1 (3)	C1—C8—C9—C14	30.1 (4)
N1—C1—C8—C9	−116.8 (3)	C10—C9—C14—C13	1.6 (4)
N4—C16—C21—C20	179.2 (3)	C10—C11—C12—C13	2.3 (6)
N4—C16—C17—C18	−179.9 (5)	C24—C23—C28—C27	0.6 (4)
N4—C15—N3—C21	−0.5 (3)	C24—C25—C26—C27	−0.5 (5)
N4—C15—C22—C23	63.8 (4)	C20—C21—C16—C17	−1.0 (4)
N2—C2—C7—C6	179.8 (3)	C20—C19—C18—C17	−0.8 (5)
N2—C2—C3—C4	178.6 (4)	C28—C23—C24—C25	−0.5 (5)
N2—C1—N1—C7	0.3 (3)	C28—C27—C26—C25	0.6 (5)
N2—C1—C8—C9	66.5 (3)	C13—C14—C9—C8	179.7 (4)
C9—C10—C11—C12	−1.2 (5)	C14—C9—C10—C11	−0.8 (4)
C9—C14—C13—C12	−0.5 (5)	C14—C13—C12—C11	−1.4 (5)
C21—N3—C15—C22	178.4 (3)	C11—C10—C9—C8	−179.0 (4)
C21—C16—N4—C15	0.4 (3)	C27—C28—C23—C22	−178.5 (4)
C21—C16—C17—C18	0.4 (4)	C22—C23—C24—C25	178.6 (4)

### Bis(acetato-κ2O,O')(2-benzyl-1H-benzimidazole-κN3)copper(II) Hydrogen-bond geometry (Å, º)

*Cg*3, 5, 8 and 9 are the centroids of the N1/C1/N2/C2/C7, C2–C7, C23–C28 and N1/C1/N2/C2–C7 rings, respectively

**Table:**

*D*—H···*A*	*D*—H	H···*A*	*D*···*A*	*D*—H···*A*
N2—H2···O4^i^	0.86 (1)	1.91 (1)	2.708 (4)	153 (1)
N4—H4···O2^ii^	0.86 (1)	1.86 (1)	2.699 (4)	164 (1)
C8—H8*a*···*Cg*3^i^	0.97 (1)	2.70 (1)	3.592 (4)	154 (1)
C8—H8*a*···*Cg*9^i^	0.97 (1)	2.93 (1)	3.844 (4)	158 (1)
C13—H13···*Cg*5^iii^	0.93 (1)	2.82 (1)	3.614 (4)	145 (1)
C30—H30*a*···*Cg*8^iv^	0.96 (1)	2.93 (1)	3.690 (5)	137 (2)

Symmetry codes: (i) −*x*+1, −*y*+1, −*z*+1; (ii) −*x*+3/2, *y*−1/2, −*z*+1/2; (iii) −*x*+1/2, *y*−1/2, −*z*+1/2; (iv) −*x*+3/2, *y*+1/2, −*z*+1/2.

## References

[bb1] Babayeva, G. O., Mamatova, G. G., Ziyatov, D. A., Makhmudova, L. S. & Daminova, S. S. (2025). *Chem. Chem. Eng.* **3**, 4–8.

[bb2] Bei, F.-L., Jian, F.-F., Yang, X.-J., Lu, L., Wang, X., Razak, I. A., Shanmuga Sundara Raj, S. & Fun, H.-K. (2001). *Acta Cryst.* C**57**, 45–46. 10.1107/s0108270100014876 11173393

[bb3] Bourhis, L. J., Dolomanov, O. V., Gildea, R. J., Howard, J. A. K. & Puschmann, H. (2015). *Acta Cryst.* A**71**, 59–75. 10.1107/S2053273314022207 PMC4283469 25537389

[bb4] Bruker (2014). *APEX2* and *SAINT*. Bruker AXS Inc., Madison, Wisconsin, USA.

[bb5] Dolomanov, O. V., Bourhis, L. J., Gildea, R. J., Howard, J. A. K. & Puschmann, H. (2009). *J. Appl. Cryst.* **42**, 339–341.

[bb6] Groom, C. R., Bruno, I. J., Lightfoot, M. P. & Ward, S. C. (2016). *Acta Cryst.* B**72**, 171–179. 10.1107/S2052520616003954 PMC4822653 27048719

[bb7] Imomov, R. B., Yusupov, Z. N. & Radjabov, U. R. (2008). *Dokl. Akad. Nauk Resp. Tajik.* **51**, 5–9.

[bb8] Jahn, H. A. & Teller, E. (1937). *Proc. R. Soc. Lond. A* **161**, 220–235.

[bb9] Krause, L., Herbst-Irmer, R., Sheldrick, G. M. & Stalke, D. (2015). *J. Appl. Cryst.* **48**, 3–10. 10.1107/S1600576714022985 PMC4453166 26089746

[bb10] Liu, L.-N., Zhang, S.-W., Wang, Y.-D., Guo, X.-G., Wu, L. & Wu, B.-L. (2014). *Inorg. Chim. Acta* **423**, 176–183.

[bb11] Lu, H.-J., Gao, J., Fan, Y.-T. & Hou, H.-W. (2003). *J. Coord. Chem.* **56**, 1025–1032.

[bb12] Lü, L. R., Tang, G. M., Wang, Y. T. & Ng, S. W. (2018). *J. Lumin.* **199**, 200–209.

[bb19] Macrae, C. F., Sovago, I., Cottrell, S. J., Galek, P. T. A., McCabe, P., Pidcock, E., Platings, M., Shields, G. P., Stevens, J. S., Towler, M. & Wood, P. A. (2020). *J. Appl. Cryst.* **53**, 226–235. 10.1107/S1600576719014092 PMC6998782 32047413

[bb13] Ohta, S., Iwabuchi, Y., Mukai, R., Ishizaki, M., Toda, T., Kurihara, M. & Okazaki, M. (2020). *Cryst. Growth Des.* **20**, 4046–4053.

[bb14] Oliynyk, S. & Oh, S. (2012). *Biomol. Ther.* **20**, 446–456. 10.4062/biomolther.2012.20.5.446 PMC3762282 24009833

[bb15] Siddikova, K., Sardor, M., Tojiboyev, A., Kadirova, Z., Ashurov, J. & Daminova, S. (2024). *Acta Cryst.* E**80**, 1186–1189. 10.1107/S2056989024008958 PMC11660480 39712173

[bb16] Spackman, P. R., Turner, M. J., McKinnon, J. J., Wolff, S. K., Grimwood, D. J., Jayatilaka, D. & Spackman, M. A. (2021). *J. Appl. Cryst.* **54**, 1006–1011. 10.1107/S1600576721002910 PMC8202033 34188619

[bb17] Tojiboyeva, I., Murodov, S., Makhmudova, L., Ziyatov, D., Ashurov, J. & Daminova, S. (2025). *Acta Cryst.* E**81**, 948–953. 10.1107/S2056989025008011 PMC12498052 41059329

[bb18] Youngme, S., Pakawatchai, C., Fun, H. K. & Chinnakali, K. (1998). *Cryst. Res. Technol.* **33**, 1586–1588.

